# Convalescent Plasma Therapy, Therapeutic Formulations of Repurposed Drugs in 20th Century Epidemics against COVID-19: A Systematic Review

**DOI:** 10.3390/pharmaceutics14051020

**Published:** 2022-05-09

**Authors:** Diego Fernández-Lázaro, Carlos Domínguez Ortega, Nerea Sánchez-Serrano, Fahd Beddar Chaib, David Jerves Donoso, Elena Jiménez-Callejo, Saray Rodríguez-García

**Affiliations:** 1 Department of Cellular Biology, Histology and Pharmacology, Faculty of Health Sciences, University of Valladolid, Campus of Soria, 42003 Soria, Spain; 2 Neurobiology Research Group, Faculty of Medicine, University of Valladolid, 47005 Valladolid, Spain; 3 Heamtology Service of the Santa Bárbara Hospital, Castille and Leon Health (SACyL), 42003 Soria, Spain; cdominguezo@saludcastillayleon.es; 4 Microbiology Unit of the Santa Bárbara Hospital, Castille and Leon Health (SACyL), 42003 Soria, Spain; 5 Department of Anatomy and Radiology, Faculty of Health Sciences, University of Valladolid, Campus of Soria, 42003 Soria, Spain; fahd.beddar@uva.es (F.B.C.); davidjerves@hotmail.es (D.J.D.); 6 Emergency Service of the Santa Bárbara Hospital, Castille and Leon Health (SACyL), 42003 Soria, Spain; 7 Neumology Service of the Santa Bárbara Hospital, Castille and Leon Health (SACyL), 42003 Soria, Spain; 8 Preventive Medicine Service of the Santa Bárbara Hospital, Castille and Leon Health (SACyL), 42003 Soria, Spain; ejimenezca@saludcastillayleon.es; 9 Department of Medicine, Faculty of Health Sciences, University of Valladolid, Campus of Soria, 42003 Soria, Spain; saray.rodriguez@uva.es; 10 Internal Medicine Service of the Santa Bárbara Hospital, Castille and Leon Health (SACyL), 42003 Soria, Spain

**Keywords:** SARS-CoV-2, COVID-19, convalescent plasma, plasma therapy, immune tool, viral load, clinical biomarkers, mortality

## Abstract

Coronavirus 2019 disease (COVID-19) represents one of the largest pandemics the world has faced, and it is producing a global health crisis. To date, the availability of drugs to treat COVID-19 infections remains limited to supportive care although therapeutic options are being explored. Some of them are old strategies for treating infectious diseases. convalescent plasma (CP) therapy has been used successfully in other viral outbreaks in the 20th century. In this study, we systematically evaluated the effect and safety of CP therapy on hospitalized COVID-19 patients. A structured search was conducted following the Preferred Reporting Items for Systematic Review and Meta-Analyses (PRISMA) guidelines using Medline (PubMed), SciELO, Cochrane Library Plus, Web of Science, and Scopus. The search included articles published up to January 2022 and was restricted to English- and Spanish-language publications. As such, investigators identified six randomized controlled trials that met the search criteria. The results determined that in hospitalized COVID-19 patients the administration of CP therapy with a volume between 200–500 mL and a single transfusion performed in 1–2 h, compared to the control group, decreased viral load, symptomatology, the period of infection, and mortality, without serious adverse effects. CP did influence clinical outcomes and may be a possible treatment option, although further studies will be necessary.

## 1. Introduction

Coronavirus disease 2019 (COVID-19) represents one of the largest pandemics facing the world, and it is producing a global health crisis [1]. COVID-19 produces a wide range of clinical symptoms, from an asymptomatic form or with mild symptoms such as cough, headache, dizziness, or fever, to the development of more severe symptoms such as viral pneumonia. The latter can be associated with respiratory failure and acute respiratory distress syndrome (ARDS), related to severe acute respiratory syndrome coronavirus 2 (SARS-CoV-2) infection and an inflammatory state [2]. This situation could lead to multiorgan failure and death [3]. The current availability of drugs to treat COVID-19 infections remains limited to supportive treatments. These are the mainstay of care, such as supplemental oxygen and mechanical ventilation in severe and critical cases. That is, treatment is generally symptomatic and manages complications [4]. Drugs such as antimalarials, antibiotics, broad-spectrum antivirals, and monoclonal antibodies have been reused. However, there are still inconclusive data on the efficacy of antivirals such as ribavirin, oseltamivir, favipiravir, and the antitumor drug plitidepsin [4]. Tocilizumab (Interleukin (IL)-6 inhibitor) appears with the ability to control the “*cytokine storm*” and reduce pro-inflammatory biomarkers with subsequent resolution of severe COVID-19 disease [5]. All currently approved or licensed COVID-19 vaccines are safe and effective and reduce the risk of becoming seriously ill. Vaccination can reduce the spread of the disease and help protect those who are vaccinated and the people around them [6]. However, like other vaccines, they are not 100% effective; some of those who are fully vaccinated will become infected with COVID-19, although most people who become ill with COVID-19 are not vaccinated [7]. By 27 January 2021, there were 364,075,086 confirmed cases of COVID-19, including 5,631,304 deaths worldwide, and a total of 9,890,987,656 vaccine doses had been administered [8]. Therefore, with the proliferation of infections worldwide, treatment strategies that are feasible and effective in dealing with the disease are urgently needed, especially in severe cases of COVID-19 [9].

In the face of the health emergency/pandemic that remains in place, healthcare must repurpose existing drugs, especially those used in previous coronavirus epidemics, such as Severe Acute Respiratory Syndrome (SARS) and Middle East Respiratory Syndrome Coronavirus (MERS-CoV) [10]. In this regard, convalescent plasma (CP) therapy has been used with relative efficacy in the treatment of SARS [11] and MERS [12]—because of the similarity of virological and clinical features between SARS, MERS, and COVID-19 [13], adding the absence of a fully effective drug [4], CP therapy may be a treatment with potential against COVID-19 [4]. CP therapy has been an immunization strategy since the 20th century in the emergency intervention of the *Spanish flu* (1917–1918), West Nile virus, 2009 influenza A (H1N1), avian influenza A (H5N1), SARS (2003), Argentine hemorrhagic fever (1960s) and the Ebola virus outbreak in the West Africa pandemics (2013–2015). Early use of a treatment with potential against COVID-19 CP therapy achieved significantly decreased case fatality rates and associated minor adverse effects [14]. In fact, CP therapy achieved a reduction in mortality in respiratory tract viral infections (Influenza and SARS-CoV) [15]. Apheresis processes allow for the obtaining of healing factors from immunized blood from donors including (actively immunized survivors with completed infections or convalescent persons) neutralizing antibodies (NAbs), cytokines with anti-inflammatory properties, key factors of the coagulation pathway, natural antibodies, defensins, pentraxins and other nonspecific proteins [16]. CP therapy would potentially neutralize the pathogen for eradication by Nabs and would also provide a positive effect against SARS-CoV-2 beyond NAbs [15]. The immunomodulatory effect allows for the control of the excessive inflammation generated by SARS-CoV-2, a process that could lead to severe COVID-19 [4]. The COVID-19 patient develops a state of systemic hyperinflammation known as a “*cytokine storm*” that can be prolonged in time, causing lung damage by developing fibrosis and decreased lung function [17]. The initial results of this treatment in patients with COVID-19 showed effectiveness in terms of clinical reduction of viral load, fewer complications, as well as a reduction in mortality [18,19]. However, Pinechota et al. [20] reported unclear results on the effect of CP therapy on mortality or prolongation of time to death due to the slightest improvement in clinical symptoms. Also, there was little certainty as to whether CP therapy increases the risk of moderate to severe adverse events (allergic or respiratory). Therefore, we conducted this study to systematically analyze the evidence of the effect and safety of treatment with CP therapy using randomized controlled trials (RCTs) to confirm the usefulness of this intervention in hospitalized patients with COVID-19. By analyzing RCTs that are considered the “gold standards” for examining whether there is a cause-effect relationship between the performance of CP and potential benefits in patients [21]. In addition, the selection of RCTs decreases the risk of selection bias and helps ensure more reliable and higher quality data [22].

Our research question was defined using the PICOS model according to the standard methods proposed by the Preferred Reporting Items for Systematic Reviews and Meta-Analyses Guidelines (PRISMA) [23] as follows: Population “patients with hospitalized COVID-19 disease”; I (intervention) “treatment by convalescent plasma transfusion”; C (comparison) “same conditions with placebo, sham therapy, or no intervention”; O (outcomes) “effects on immune response, duration of infection, recovery time, hospitalization rates, disease progression to different stages, need for oxygen therapy and mortality”; These variables were included as outcomes, as they are usually investigated in studies on CP administration; S (study design) “randomized controlled trials”. The review protocol is published in the Prospective Register of Systematic Reviews (PROSPERO); ref CRD 42022314038.

## 2. Method

### 2.1. Search Strategy and Study Selection

We developed a structured search using the databases Medline (PubMed), SciELO, Cochrane Library Plus Web of Science (WOS) and Scopus, for articles published from database inception to 31 January 2022, restricted to English and Spanish language, all of which are high-quality databases which guarantee good bibliographic support. The terms used in the primary search were related to the use of convalescent plasma therapy in hospitalized COVID-19 patients and the different biomarkers of outcome. For keywords for the search we used Medical Subject Headings (MeSH),such as “convalescent plasma”, “antibodies”, “blood transfusion”, “serum immunoglobulins”, “neutralizing antibodies”, “cytokines”, “plasma therapy”, “Coronavirus disease 2019 (COVID-19)”, “SARS-CoV-2”, “coronavirus”, “acute respiratory syndrome coronavirus 2”, “patients hospitalized”, “critically ill patients”, “immune response”, “Survival rate”, “duration of infection”, “Virus shedding”, “disease progression”, “oxygen therapy”, and “mortality”; additionally, the Boolean operators “AND” and “OR” were used as a search nexus. The full search strategy is included in Appendix A. Two reviewers (D.F.-L. and N.S.-S.) independently screened titles and abstracts, and full texts were sourced for relevant articles. Inclusion criteria were independently assessed, and disagreements were resolved by a third reviewer (S.R.-G.). No additional records were obtained through reference lists of included of relevant articles.

### 2.2. Selection of Articles: Inclusion Criteria

The selection of studies was based on the following criteria: (a) adults hospitalized by COVID-19 in moderate, severe, or severe clinical situations (excluding animal and/or in vitro studies); (b) studies that evaluated the effects of administration CP; (c) randomized controlled trials (excluding editorial records, reviews, notes, and any other non-original studies); (d) studies with clear information on the administration of CP (dose, frequency, and mode); (e) studies that evaluated as outcomes were immune response, duration of infection, recovery time, hospitalization rates, disease progression to different stages, need for oxygen therapy and mortality; (f) studies with methodological quality ≥ 10 points, according to the McMaster critical review form [24] for quantitative studies. Records that did not meet the criteria were excluded from this systematic review.

### 2.3. Assessment of Methodological Quality

The methodological quality evaluation of the selected articles was assessed using McMaster’s Critical Review Form [24]. The aim of this evaluation was to exclude studies with poor methodology. The methodological quality of the selected studies was assessed by the same two authors (D.F.-L. and N.S.-S.), and any disagreements were resolved by third-party evaluation.

### 2.4. Data Extraction

Two reviewers (D.F.-L. and N.S.-S.) scrutinized and synthesized the data of all the selected studies into a comprehensive table using a standardized data extraction. Disagreements were resolved by a third reviewer (S.R.-G.). Information extracted from the selected studies included the name of the first author, year of publication, the country where the study was conducted, study design, sample size, sex and age of the participants, dosage, timing of the convalescent plasma, duration of intervention, mode of administration, outcomes, and results.

## 3. Results

### 3.1. Selection of Studies

**Figure 1:**
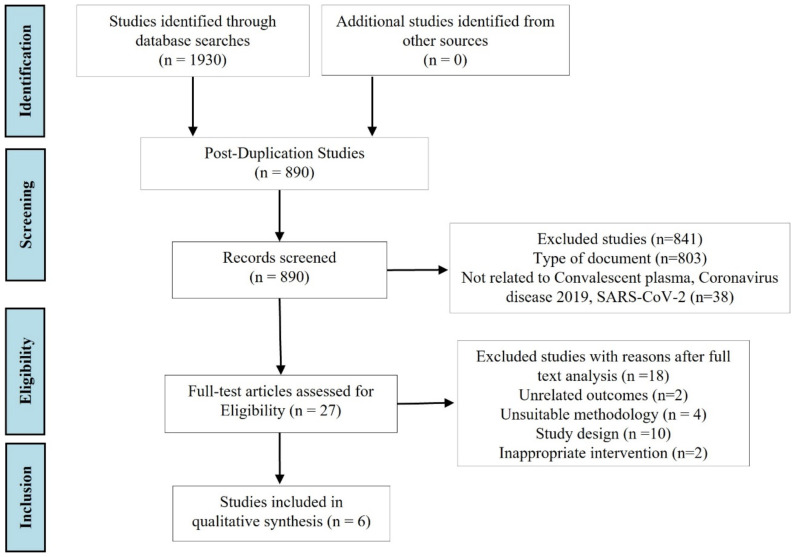
Preferred Reporting Items for Systematic Reviews and Meta-Analyses (PRISMA) flow diagram study selection process for the systematic review.

We identified an initial total of 1930 records. Among those, we removed 1040 duplicates, 803 articles by the type of document, 38 not related to convalescent plasma, coronavirus disease 2019 or SARS-CoV-2. We also excluded 18 articles after full-text review. Reasons for exclusions after full-text review were unrelated outcomes (n = 2), unsuitable methodology (n = 4), study design (n = 10) and inappropriate intervention (n = 2), and the remaining six studies [25,26,27,28,29,30] met our inclusion criteria and were included in the present systematic review (Figure 1).

### 3.2. Results of the Quality Assessment

Then we conducted the quality assessment of the articles. The score of the selected articles ranged from 10 to 13 points. Two studies were assessed as “acceptable”, three as “very good”, and one as “very good”. No studies were excluded because of poor quality. Details about the results of the quality assessment are shown in Table 1.

### 3.3. Descriptive Information of the Selected Articles Included in the Systematic Review

The characteristics of the studies included in the systematic review appear in Table 2. The total number of participants at baseline was 861, of which 368 are patients in severe/critical clinical status (dyspnea, tachypnea > 30 breaths/minute, blood oxygen saturation < 93%, PaO_2_/FiO_2_ ≤ 300 and pulmonary infiltrates of more than 50%) [25,26,29,30] and 493 in moderate status -mild-moderate pneumonia, with typical symptomatology of cough, dyspnea, and fever- [27,28]. The protocol of the interventions varied in terms of volume and frequency of administration. However, in all studies, a single dose of PC was administered [25,26,27,28,29,30]. The volume of PC administered varied between 200 mL [29] to 500 mL [28], with administration frequencies between 1–2 h [25,27], 2 h [25,27] or 100 mL/hour [29].

### 3.4. Clinical Measures

Table 3 summarizes the studies included in the present review and depicts information about the authors, publication year, country, study design, population, clinical biomarkers, results, and conclusions of the selected studies.

## 4. Discussion

CP therapy is a passive immunization mechanism whereby plasma from a patient who has recovered from acute COVID-19 contains highly specific SARS-CoV-2 antibodies [9]. This antiviral mechanism is comprised of NAbs, and protective non-NAbs such as immunoglobulins (Ig) IgG and IgM [14]. Non-NAbs that bind directly to SARS-CoV-2 do not alter replication but would have prophylactic and/or recovery action and may even limit the cytokine storm; plasma-transfected IgG neutralize cytokines such as IL-1β and TNFα and cellular damage induced by activation of the complement cascade [31,32]. A priori, CP therapy would be a treatment measure for patients severely affected by COVID-19 and may be preventive in subjects at high risk of contracting the virus due to comorbidities. Other patients at risk are potentially those who are not vaccinated or in patients who are vaccinated but unable to generate an effective immune response. Perhaps their immune system is altered by immunosuppressive treatments or an underlying disease [33]. A priori in the COVID-19 pandemic, this classical adaptive immunotherapy strategy has been reused with potential guarantees of success. In this regard, the U.S. Food and Drug Administration (USFDA) indicated that CP therapy has a potential effect against SARS-CoV-2 [34]. In addition, the National Health Commission of the People’s Republic of China clinical guidelines indicate that CP therapy would be appropriate for severe/critically ill patients with rapid progression of COVID-19 [21]. Also, the Sree Chitra Tirunal Institute of Medical Sciences and Technology (India) has licensed CP therapy for treatment of patients infected with SARS-CoV-2 [35]. In Europe, the European Medicines Agency (EMA) and Spanish Agency of Medicines and Health Products, includes CP as an available treatment subject to special access conditions for the management of SARS-CoV-2 respiratory infections [36]. In January 2022, the European Union earmarked €36 million for projects to collect convalescent plasma recovered from COVID-19. These PC donations will be used for the treatment of patients with SARS-CoV-2 infection [37]. In this systematic review, we set out to evaluate the impact of CP therapy interventions in hospitalized patients with COVID-19 by analyzing randomized controlled studies (RCTs). This study identified and summarized six RCTs that showed a decreasing trend in the period of infection, symptomatology and mortality related to SARS-CoV-2 infection, with no serious adverse effects associated with CP therapy. All patients, regardless of whether they belonged to the CP or control group (CG), received standard anti-SARS-CoV-2 treatment: oxygen therapy, corticosteroids, antibiotics, immunomodulators, antimalarials and antivirals as supportive care [25,26,27,28,29,30]. With this systematic review of PC with different administration regimens, we have described and critically analyzed all available data in published RCTs and analyzed the possible clinical implications, efficacy, and safety in hospitalized patients in severe stages. This study could be of interest because we critically evaluated the most important biomarkers in the clinical management of COVID-19 viral load, symptomatology, time to infection, and mortality. In addition, we evaluated the safety of PC.

The volume of CP used in the studies ranged from 200 mL [29] to 500 mL [28]. The differences in the quantity of CP therapy used could be due to the content of NAbs. In addition, this would condition the results on clinical biomarkers in each study included in this systematic review. The efficacy of this therapy is dependent on NAbs concentration in the recovered donor plasma [14]. NAbs in SARS-CoV and MERS block the development of viral infection by their interaction with the S1 receptor binding protein (S1-RBD), the S1-N-terminal domain, and S2. COVID-19 NAbs are unique, and this is because they block the interaction between ACE2 and the receptor binding domain of the SARS-CoV-2 spike protein [38]. However, approximately 30% of donors generated low titers of Nabs [39], but it is possible that the current tests are not sufficiently sensitive and these data will have to be validated with new generation enzyme-linked immunosorbent assays (ELISAs), which would indicate that other components of the CP therapy are responsible for its potential therapeutic effect, such as complement activation, antibody-dependent cellular cytotoxicity and/or phagocytosis, IgG and IgM [18]. The titers of anti-spike SARS-CoV-2 IgG are different in each study; the detection methods and Ig titer evaluation rates were also different, and an initial high titer would promote an earlier therapeutic effect [40]. Rasheed et al. [30] reported a significant increase in IgM and IgG at three days post CP transfusion. Also, Libster et al. [27] have reported increased IgG levels at 24 h in CP group with respect to CG; it should be considered that after CP transfusion, IgG and IgM titers increase in a time-dependent manner, which would allow greater efficacy after a short time [40]. However, there is not a standard transfusion dose of CP. We think that the optimal dose cannot be determined due to the different titers.

A single dose of intravenous transfusion of CP was administered (25–30), although the use of one or two additional doses would also be indicated depending on the severity and tolerance of the patient [18]. The frequency of intravenous transfusion of CP therapy was different in each study (Table 2). The British Society of Hematology states that administration time with plasma is 10–20 mL·kg^−1^·h^−1^, longer administration time should be considered in patients at risk of circulatory overload [31], and Jafari et al. [41] recommend administering 3 mL·kg^−1^ per dose over two days, which would allow for the distribution of 250 mL per unit and would standardize CP therapy. Therefore, differences in titers, volume and frequency of administration were found in the included studies that could influence the efficacy and safety of CP therapy treatment. The infusion of small plasma volumes (from 200 mL to 500 mL) has little bearing on the recipient’s blood concentration and on the therapeutic effect of drugs, which are individually adjusted by plasma volume, among others [42]. However, there are potential risks of drugs in the donated plasma, as these drugs may be allergenic or potentially harmful to the recipient’s blood [38]. In this way, of a total of 426 patients hospitalized with SARS-CoV-2 infection and treated with CP therapy, only three patients had adverse effects related to this treatment. Rasheed et al. [30] described that one patient suffered from mild redness and itching of the skin 1 h after treatment administration, which subsided with an intramuscular injection of an antihistamine drug. Li et al. [29] reported that one patient manifested urticaria as a mild adverse reaction, while another patient suffered a severe adverse reaction related to hypoxia/cyanosis due to an anaphylactoid reaction (anaphylactic-like reaction). In this way, CP therapy has minimal or no adverse effects [43]. It should be considered that as with any hemocomponent, CP transfusion carries the risk of adverse reactions, such as non-hemolytic febrile reactions, allergic reactions, transfusion-associated infections, hemolytic reactions, and more severe reactions such as transfusion-related acute lung injury or transfusion-associated circulatory overload [44].

There is a correlation between the development of antibodies and the disappearance of the virus in the pharynx as determined by Real-Time Reverse Transcription Polymerase Chain Reaction (RT-PCR) [43]. Li et al. [29] y Zeng et al. [26] have described that the disappearance of the viral load is significantly higher in the group that received the CP therapy compared to the CG. In addition, viral shedding is reduced in the CP group in critically/severe patients with respiratory failure in non-early stages of the disease, the duration of illness is significantly shorter, and hospital discharges show a positive trend in patients who received CP therapy [26]. In addition, improved clinical biomarkers at 14- and 28-days post-treatment and hospital discharge rates at 28 days [29], significantly decreased the duration of infection and recovery time [30]; in addition, a significant reduction in the risk of developing severe or critical respiratory disease was shown [27]. These are some of the positive results obtained in patients who were administered CP therapy. However, the requirement for invasive ventilatory support, oxygen therapy, time to discharge with full return to baseline physical function, and mortality rates are not changed by the administration of CP therapy, although there is a trend toward improvement in hospital discharge without full return to baseline physical function [28]. This could be due to insufficient neutralizing activity, given that around 50–70% of patients have medium or medium/low neutralizing activity in plasma and only 1–5% of patients generate high neutralizing titers [45].

In COVID-19, viremia peaks in the first week of infection and viral loads are highly correlated with disease severity and progression [4]. Early administration of CP therapy could reduce symptoms, progression to more severe stages and even death from virus-related damage [16]. Therefore, the ability to reduce mortality in this situation of health emergency after almost six million deaths makes it an essential parameter to evaluate the efficacy of CP therapy. Five studies [25,26,27,29,30] showed a reduction in mortality after the administration of CP, two of them significantly [26,30], therefore it could be considered that early administration of CP could contribute to mortality reduction. This is similar to the findings reported by Cheng et al. [11], who observed lower mortality when administering CP in the first 14 days of COVID-19. In addition, Liu et al. [25] have reported that the probability of survival is significantly higher in the CP therapy group, which could be related to the significant reduction in the rate of worsening blood oxygen saturation or the reduced need for oxygen therapy administration. Although mortality in patients with noninvasive ventilation was lower in patients with CP therapy with respect to CG, there were no differences in patients with invasive ventilation [25]. Similarly, the use of CP in combination with standard treatment decreases mortality in patients with severe disease versus critically ill patients, and patients treated early (within the first days of hospitalization) at elevated neutralizing antibody titers [46,47]. However, PC for severe/critical ill patients with COVID-19 remains controversial. Some studies [48,49,50] found that this CP did not achieve good clinical outcomes, mainly related to mortality reduction. This could be due to several differences, such as inadequate IgG titer of CP (unknown on some studies), the standard anti-SARS-CoV-2 treatment used for the CP group and the control group was not the same, the PC was used as a single therapy; PC administration was given in late post-infection phases of COVID-19; high age profile, numerous comorbidities, and a different viral load of patients. Also, it should be considered that the efficacy of PC also depends on several curative factors present in CP [16].

## 5. Conclusions

CP has the potential to provide a promising and immediate treatment option because it decreases viral load, symptomatology, time to infection and mortality without serious adverse effects. Thus, CP takes on a key role while existing drugs are being evaluated and new vaccines and targeted therapies are being developed. Moreover, by not relying solely on the neutralizing activity of NAbs, it could be an additional protective mechanism against different viral variants. in addition, it has recently been described that the humoral response to mRNA vaccines (Comirnaty^®^ or Spikevax^®^) was severely impaired in patients undergoing B-cell targeted therapies (either Rituximab or Ibrutinib) [51]. Adverse effects are mild, easily controllable and of lesser entity than other plasma-derived treatments.

However, results could be influenced by administration time, dose, titer, small sample size, and participant characteristics such as age, gender, ethnicity, and COVID-19 stage. At the same time, the affinity and specificity characteristics of the antibodies present in CP are highly variable and difficult to standardize, which can affect efficacy and hinder the interpretation of assays aimed at assessing their therapeutic action. It must be considered that the efficacy of CP depends on the mechanisms of the patient’s immune system, which may be altered during COVID-19. Consequently, it is difficult to draw firm conclusions about whether CP therapy in hospitalized patients with COVID-19 is effective. Thus, this systematic review demonstrates the need for further studies to reevaluate the effects of CP therapy as a passive immunization strategy in patients infected with SARS-CoV-2 to determine possible improvements in clinical biomarkers, the safety profile, and patient health status. Thus, we suggest that at this time, CP for hospitalized patients with COVID-19 should not be used outside of RCTs. Despite these limitations, the strengths of this systematic review are based on the use of the PRISMA guidelines [23] and the McMaster Quantitative Review Form [24].

## Figures and Tables

**Table 1:** Quality assessment of the studies included in the systematic review.

Author/s	Items																T1	%	MQ
	1	2	3	4	5	6	7	8	9	10	11	12	13	14	15	16			
Rasheed et al. [30]	1	1	1	0	1	1	1	1	0	1	1	1	1	1	0	1	13	81.25	VG
Li et al. [29]	1	1	1	1	1	1	0	1	0	1	0	1	1	1	1	0	12	75	G
Simonovich et al. [28]	1	1	1	1	0	1	1	1	1	0	0	1	1	1	0	1	12	75	G
Libster et al. [27]	0	1	1	1	0	0	1	1	0	0	0	1	1	1	1	1	10	62.5	A
Zeng et al. [26]	0	1	1	1	0	1	1	0	1	1	1	1	1	0	0	1	11	68.75	G
Liu et al. [25]	1	1	1	1	0	0	1	1	0	0	0	1	1	1	0	1	10	62.5	A
T2	4	6	6	5	2	4	5	5	2	3	2	6	6	5	2	5			

Abbreviations: (1) Criterion was met; (0) Criterion was not met; (T1) Total items fulfilled by study; (T2) Number of studies fulfilled the item; (%) Percentage of methodological quality assessment; (MQ) Methodological Quality; (A) acceptable 9–10 points; (G) good 11–12 points; (VG) very good 13–14 points.

**Table 2:** Descriptive synthesis of the studies included in the systematic review.

Characteristics	Type	Study Reference
Clinical status of hospitalized patient COVID-19	moderate	[27,28]
	critical/severe	[25,26,29,30]
Quantity/Volume of convalescent plasma with positive immunoglobulins G (IgG+)	500 mL	[28]
	400 mL	[30]
	300 mL	[26]
	250 mL	[25,27]
	200 mL	[29]
Dose of intravenous transfusion of convalescent plasma	Single dose	[25,26,27,28,29,30]
Frequency of intravenous transfusion of convalescent plasma	Continued in 2 h	[30]
	10 mL (first 15 min)–100 mL/hour	[29]
	Continuous between 1–2 h	[25,27]

Moderate hospitalized patient COVID-19: moderate pneumonia, with typical symptomatology of cough, dyspnea, and fever; critical/severe hospitalized patient COVID-19: dyspnea, tachypnea > 30 breaths/minute, blood oxygen saturation < 93%, PaO_2_/FiO_2_ ≤ 300 and pulmonary infiltrates of more than 50%; mL: milliliters.

**Table 3:** Summary of the results of the studies included in the systematic review.

Author/s—Year—Country	Study Design	Population	Clinical Biomarkers	Results CP vs. CG	Main Conclusions
Rasheed et al. [30] 2020 Iraq	Randomized controlled clinical trial	*Hospitalized patient COVID-19:* Clinical status: critical n = 49 → CP = 21; CG = 28 Age: 47–56 years *Plasma Donors:* <50 years; healthy habits; non-pregnant; without comorbidities and recovered from COVID-19 (two negative tests: test two weeks; test two days before donation). IgG ≥ 1.25 of anti-SARS-CoV-2 IgG antibodies	IgM (day three)	↑*	Convalescent plasma therapy is an effective mode of therapy if donors with high level of SARS-CoV-2 antibodies are selected and if recipients were at their early stage of critical illness
			IgG (day three)	↑*	
			Recovery time	↓*	
			Duration of infection	↓*	
			Death rate	↓*	
			Adverse Events	↔	
Li et al. [29] 2020 China	Randomized controlled clinical trial	*Hospitalized patient COVID-19:* Clinical status: severe/critical CP: n = 23 severe/29 critical CG: n = 22 severe/29 critical n = 103 → CP: n = 52; CG: n = 51 Age: 62–78 years; Sex: 60♂; 43♀ *Plasma Donors:* Age: 18–55 years; two negative CPR test results, two weeks from hospital discharge, no Long-COVID-19 symptoms S-RBD–specific IgG ≥1:640 titer	Rate of clinical improvement	Day 7: ↔ Day 14:↑ Day 28: ↑	Convalescent plasma therapy added to standard treatment, compared with standard treatment alone, did not result in a statistically significant improvement in time to clinical improvement within 28 days
			Hospital discharge rate	Day 28: ↑	
			Mortality at 28 days	↓	
			Negative rate of SARS-CoV-2 nucleic acid	24 h: ↑ 48 h: ↑ 72 h: ↑*	
			Adverse Events	↑	
Simonovich et al. [28] 2020 Argentina	Randomized controlled clinical trial → Multicenter & double-blind	*Hospitalized patient COVID-19:* Clinical status: mild/moderate n = 333 → CP: n = 228; Age: 52–73.5 years; Sex:67♀ 161♂ CG: n = 103; Age: 49–71 years; Sex: 41♀ 62♂ *Plasma Donors:* A single donor or a pool of two to five donors Specific SARS-CoV-2 IgG ≥ 1:800 titer	Need for invasive ventilatory support	↔	No significant differences were observed in clinical status or overall mortality between CP and CG. However, CP was a trend towards improvement but without full recovery of baseline physical function.
			Oxygen requirement	↔	
			Individuals at discharge with full return to baseline physical function	↔	
			Discharge without full return to baseline physical function	↑	
			Time from intervention to clinical improvement	↔	
			Mortality	↔	
			Adverse Events	↔	
Libster et al. [27] 2020 Argentina	Randomized controlled clinical trial → double-blind	*Hospitalized patient COVID-19:* Clinical status: mild/moderate n = 160 → CP: n = 80; Age: 76.4 ± 8.7 years; Sex: 68%♀ 32%♂ CG: n = 80; Age: 77.9 ± 8.4 years; Sex: 58%♀ 42%♂ *Plasma Donors:* n = 135; SARS-CoV-2 S IgG ≥ 1:1000 titer, Patients SARS-CoV-2 infection ≥10 days and have been asymptomatic for at least three days with two negative PCR tests	Development of severe respiratory disease	↓*	Early administration of convalescent plasma of titer ≥ 1:1000 against SARS-CoV-2 to mildly infected older adults reduced the progression of COVID-19 and could stimulate recovery of at-risk patients.
			Development of critical respiratory illness	↓	
			IgG (at 24 h)	↑	
			Development of critical systemic disease	↔	
			Mortality	↓	
			Adverse Events	↔	
Zeng et al. [26] 2020 China	Randomized controlled clinical trial	*Hospitalized patient COVID-19:* Clinical status: severe/critical 100% ICU admission n = 21→ CP: n = 6; Age: 61.5 years (31.5–77.8); Sex:1♀ 5♂ CG: n = 15; Age 73 years (60–79); Sex: 4♀ 11♂ *Plasma Donors:* Recovered from COVID-19 1 or two weeks ago, two negative COVID-19 tests, SARS-CoV-2 IgM−/IgG+ by gold immunochromatography tests (Innovita Biotech).	Duration of COVID-19	↓*	CP therapy could stop SARS-CoV-2 shedding and extend survival in patients with COVID-19. However, cannot reduce the mortality rate in critically ill patients with end-stage disease. CP therapy in critically ill patients with COVD-19 early in the course of disease
			Viral spread	↓	
			Hospital discharges	↑	
			Viral load disappearance	↑*	
			Mortality	↓	
			Adverse Events	↔	
Liu et al. [25] 2020 USA	Randomized controlled clinical trial	*Hospitalized patient COVID-19:* Clinical status: severe/critical Life-threatening COVID-19 → medical criteria n = 195 → CP: n = 39; Age: 55 ± 13 years; Sex: 25♂ 14♀; BMI: 31.7 ± 6 Kg·m^−2^ CG: n = 156 *Plasma Donors:* n = 25 Recovered from COVID-19, 2 negative COVID-19 tests, anti-spike SARS-CoV-2 IgG ≥ 1:320 titers	Worsening rate of oxygenation (at 14 days)	↓*	CP significantly increases survival. CP is most effective in the early phase of the disease with no significant difference between non-intubated and intubated recipient patients
			Need for oxygen therapy	↓	
			Probability of survival	↑*	
			Death rate in patients with noninvasive ventilation	↓	
			Death rate in patients with invasive ventilation	↔	
			Adverse Events	↔	

CP: convalescent plasma group; CG: control group; ♂: male; ♀: female; *n*: total number of participants; ↑: increase; ↓: decrease; ↑*: significant increase; ↓*: significant decrease; ↔: no change; IgG: immunoglobulin G; IgM: immunoglobulin M; BMI: body mass index; PCR: polymerase chain reaction. S-RBD: IgG antibodies directed against the RBD domain of the S1 subunit (spicule); COVID-19: Coronavirus disease 2019; SARS-CoV-2: severe acute respiratory syndrome coronavirus 2.

## Data Availability

Not applicable.

## Appendix A. Search Terms Used for the Selection of Articles

#1 “convalescent plasma”[Mesh]
#2 “antibodies”[tiab] OR “blood transfusion”[tiab] OR “immunoglobulins”[tiab] OR “neutralizing antibodies”[tiab] OR “cytokines”[tiab] OR “plasma therapy” [tiab]
#3 #1 OR #2
#4 “COVID-19”[Mesh]
#5 “SARS-CoV-2”[tiab] OR “coronavirus”[tiab] OR “Coronavirus disease 2019”[tiab] OR “acute respiratory syndrome coronavirus 2”[tiab]
#6 #4 OR #5
#7 “patients hospitalized”[Mesh]
#8 “critically ill patients”[tiab] OR “immune response”[tiab] OR “Survival rate”[tiab]OR “duration of infection”[tiab] OR “Virus shedding”[tiab] OR “disease progression”[tiab] OR “oxygen therapy”[tiab] OR “mortality”[tiab]
#9 #7 OR #8
#20 #3 AND #6 AND #9 AND

The search was conducted through Medline (PubMed), SciELO, Cochrane Library Plus Web of Science (WOS) and Scopus for articles published from database inception to 31 January 2022, restricted to English and Spanish language.
